# The genotypic and phenotypic characteristics contributing to high virulence and antibiotics resistance in *Escherichia coli* O25-B2-ST131 in comparison to non- O25-B2-ST131

**DOI:** 10.1186/s12887-023-03866-w

**Published:** 2023-02-03

**Authors:** Razieh Shahbazi, Siavosh Salmanzadeh-Ahrabi, Mohammad Mehdi Aslani, Masoud Alebouyeh, Jamal Falahi, Vajihe Sadat Nikbin

**Affiliations:** 1 Department of Microbiology, Faculty of Biological Sciences, Alzahra University, Deh Vank Ave., Tehran, 1993891176 Iran; 2 Department of Bacteriology, Pasteur Institute of Iran, Tehran, Iran; 3 Pediatric Infections Research Center, Research for Children’s Health, Shahid Beheshti University of Medical Sciences, Tehran, Iran; 4 Health Clinical Science Research Center, Zahedan Branch, Islamic Azad University, Zahedan, Iran

**Keywords:** *Escherichia coli*, ST131, UTI, *Bla* gene; rep-PCR

## Abstract

**Background:**

*Escherichia coli* serogroup O25b*-*sequence type 131 (*E. coli* O25-B2-ST131) is considered as multidrug-resistant and hypervirulent organism. There is lack of data about involvement of this pathogen in the children’s infection. In this study, the prevalence, and clonality, virulence capacity, and antibiotic resistance phenotype and genotype of *E. coli* O25-B2-ST131 compared with non-O25-B2-ST131 isolates were investigated in children with urinary tract infection in Tehran, Iran.

**Methods:**

The *E. coli* isolates from urine samples were identified using conventional microbiological methods. Characterization of *E. coli* O25-B2-ST131 clone, antibiotic susceptibility, biofilm formation, ESBLs phenotype and genotype, serum resistance, hemolysis, hydrophobicity, and formation of curli fimbriae were done using conventional microbiological and molecular methods. Clonality of the isolates was done by rep-PCR typing.

**Results:**

Among 120 *E. coli* isolates, the highest and lowest antibiotic resistance was detected against ampicillin (92, 76.6%) and imipenem 5, (4.1%), respectively. Sixty-eight (56.6%) isolates were ESBL-producing and 58 (48.3%) isolates were considered as multi-drug resistance (MDR). The prevalence of ESBL-producing and MDR isolates in O25-B2-ST131 strains was higher compared with the non-O25-B2-ST131 strains (*p* value < 0.05). O25-B2-ST131 strains showed significant correlation with serum resistance and biofilm formation. Amongst the resistance and virulence genes, the prevalence of *iucD, kpsMTII, cnf1, vat*, *bla*_CTX-M-15,_ and *bla*_SHV_ were significantly higher among O25-B2-ST131 isolates in comparison with non-O25-B2-ST131 isolates (*p* value < 0.05). Considering a ≥ 80% homology cut-off, fifteen different clusters of the isolates were shown with the same rep-PCR pattern.

**Conclusions:**

Our results confirmed the involvement of MDR-ESBLs producing *E. coli* strain O25-B2-ST131 in the occurrence of UTIs among children. Source tracking and control measures seem to be necessary for containment of the spread of hypervirulent and resistance variants in children.

## Background

*Escherichia coli* is an extremely variable Gram-negative bacterium with the potential to colonize in different animals and humans. However, *E. coli* is categorized as a nonpathogenic microorganism and is part of the normal intestinal flora of animals and humans [1]. Some pathogenic *E. coli* can cause gastrointestinal and extra-intestinal infections. Extra-intestinal pathogenic *E. coli* possesses an intricate phylogeny and a substantial genome plasticity. Moreover, infection with this bacterium leads to various diseases such as uncomplicated urinary tract infections (UTIs), septicemia, life-threatening blood stream infections and peritonitis [2]. Uropathogenic *E. coli* (UPEC) is considered as a highly uropathogen causing acute community-acquired urinary tract infection (UTI), and is responsible for 80 to 90% of UTIs in children [1, 3]. UPEC strains have specific virulence factors that account for the progression and development of the disease. Recurrent infection and antibiotic resistance make *E. coli* an interesting subject for microbiological investigations [4]. It has been emphasized that different *E. coli* phylogenetic groups significantly affect the bacterial pathogenicity. Extraintestinal pathogenic strains mainly belong to group B2 and to a lesser extent to group D, which are often clonal [5]. The presence of *bla*_CTX-M-15_ as a common type of ESBLs was responsible for the global outbreak of ESBL-producing *E. coli*, and notably, this type often belongs to a sequence type (ST) called ST131. Studies focusing on children showed that this sequence type (ST) of *E. coli* is also responsible for infections in humans. It is accounted for 8% of urinary non-ESBL-producing isolates in children from Australia and 10.2% of urinary CTX-M-producing *E. coli* isolates from children in Texas Children's Hospital (USA) [6]. The epidemic clones of *E. coli* ST131 carry a high number of virulence and resistance genes. Not only resistance to routine antibiotics was reported in *E. coli* ST131, but also resistance to carbapenems and colistin were a matter of great concern in this strain (de la Tabla et al., 2017, Ripabelli et al., 2020, [7]. Therefore, the O25-B2-ST131 clonal group is considered as an international multi-drug resistance (MDR) high-risk clone. The clone type is associated with a broad spectrum of infections, such as intra-abdominal, bloodstream, and soft tissue infections, as well as septic shock, epididymo-orchitis, and meningitis [8]. However, the recently emerged *E. coli* ST131 clone plays a significant role in community- and hospital-acquired UTIs [9]. UTIs are considered as a severe public health problem, approximately seven times more among the females compared to the males [1, 10]. Community-acquired UTIs (CA-UTIs) are considered as public health issue. Depending on the microbial etiologies, the disease could vary from asymptomatic to debilitating, which necessitates its early diagnosis and treatment. Among common pathogens associated with CA-UTI, the role of *E. coli* O25-B2-ST131 as a hypervirulent variant is not fully known. On the other hand, the level of antibiotic resistance, virulence factors, colonization, and spread in a variety of niches of this bacterium varies in different parts of the world and there is a need for detailed studies [2, 11]. To understand involvement of the *E. coli* O25-B2-ST131 in the occurrence of UTIs in children, this study was aimed to investigate prevalence of this clonal group among among children with UTI. Moreover, homology of the isolates, their virulence capacity, resistance to antimicrobials and carriage of genes related to ESBLs phenotype were investigated in these isolates compared with non-O25-B2-ST131 *E. coli* isolates.

## Methods

### Bacterial isolates

A total of 120 non-duplicate clinical isolates of *E.coli* were collected from outpatients (children aged 2 to 7 years) with UTI who referred to Mofid Hospital from May to September 2019. All samples were transferred to the microbiology laboratory and clinical *E.coli* isolates were identified using conventional biochemical tests. The *E.coli* isolates were stored in a TSB medium containing 10% glycerol at -20 °C for further examinations [12].

### Antimicrobial susceptibility testing

Antimicrobial susceptibility testing was performed using the BD (New Jersey, USA) and Mast (Liverpool, UK) antibiotic disks according to the Clinical and Laboratory Standards Institute (CLSI, 2019). The antibiotics used for disk diffusion method were included azitromycin, ampicillin, cefazolin, cefoxitin, cefotaxime, ceftazidime, cefepime, ciprofloxacin, imipenem, nalidixic acid, gentamicin, amikacin, aztreonam, tetracycline, ampicillin sulbactam, amoxicillin-clavulanic acid, piperacillin tazobactam, meropenem, nitrofurantoin, and trimethoprim + sulfamethoxazole [13]. On the other hand, based on the antibiotic resistance pattern, we examined the prevalence of MDR isolates according to Magiorakos et al. [14, 15].

### Minimum inhibitory concentration (MIC) of colistin

The lyophilized powder of colistin sulfate salt was purchased from Sigma-Aldrich (Merck, Germany) and was re-suspended in distilled sterile water. A final concentration of colistin vials (1,024 μg/ml) was stored at − 80 °C for further tests. In addition, Mueller–Hinton broth was prepared in separate tubes for different concentrations of colistin, ranging from 0.5 to 16 mg/L with two-fold dilutions according to the recommendations by CLSI/EUCAST guidelines. The *E. coli* isolates with MIC ≥ 4 mg/mL of colistin were determined as resistant. For each isolate tested, a positive and negative control were included in the first and second wells of the plate, respectively. *E. coli* ATCC 25,922 was used as a standard control [16].

### Screening of ESBL phenotype

ESBL-producing *E. coli* isolates were characterized by double-disk synergy test, which was carried out using cefotaxime-clavulanic acid and ceftazidime-clavulanic acid as a two-disc synergism versus ceftazidime and cefotaxime alone. The increased inhibition zone ≥ 5 mm around the discs with clavulanic acid assigned the bacterium as ESBL-producing. Furthermore, *Klebsiella pneumoniae* ATCC 700,603 and *E. coli* ATCC 25,922 were considered as positive and negative controls, respectively [17].

### Serum resistance and hemolysin production

The resistance of *E. coli* isolates to killing by pooled serum and production of hemolysin was assessed by the method defined by Montenegro et al., with few modifications [18]. In the serum resistance test, equal volumes of serum and bacterial suspension were blended to obtain an ultimate serum concentration of 50% (v/v). The mixture was incubated for 120 min.

### Surface hydrophobicity and curli fimbriae production

Surface hydrophobicity was assessed using the salt aggregation test (SAT) [19] and the production of curli fimbriae by *E. coli* isolates was determined via culture on a salt-free LB agar plate containing congo red and brilliant blue dyes. Finally, the presence or absence of fimbriae was determined based on morphotypes [20].

### *In vitro *biofilm formation assay

Microtitre plate method was performed as the gold standard quantitative method for characterizing biofilm-forming *E. coli* isolates. Briefly, overnight culture of *E. coli* isolates was inoculated into trypticase soy broth (3 mL, Merck, Germany), supplemented with 1% glucose, and incubated at 37 °C for 24 h. Afterward, the culture was diluted at 1:100 by adding sterile trypticase soy broth and 200 mL of dilution was added to each well of a sterile 96-well polystyrene microtiter plate. Three wells for each isolate were assessed in each microtiter plate. This pattern was repeated in three microtiter plates. The plates were covered and incubated aerobically for 24 h at 37 °C, and subsequently, washing (250 μL of sterile saline solution), fixing (200 μL of methanol), staining (200 μL 2% Hucker crystal violet per well), and drying were performed. Finally, a micro-enzyme-linked immunosorbent assay (ELISA) reader determined the absorbance of biofilm formation at 570 nm. Then, 200 mL of sterile TSB was inoculated in wells as negative control. Biofilm-forming isolates were categorized in four groups, including strongly adherent bacteria (4 × ODc < OD), moderately adherent bacteria (2 × ODc < OD ≤ 4 × ODc), weakly adherent bacteria (ODc < OD ≤ 2 × ODc), and non-adherent bacteria (OD ≤ ODc) [21, 22].

### Genomic DNA preparation

To extract the genomic DNA of *E. coli*, each isolate was grown overnight in Luria–Bertani broth (Merck, Germany) medium at 37 °C, and 1 ml broth culture was harvested by centrifugation at 8000 rpm for 10 min. Total bacterial DNA from each sample was extracted by the boiling and freezing methods. The quality of the extracted DNA was confirmed by measuring absorbance (A_260/_A_280_) by a NanoDrop (Thermo Scientific, Roskilde, Denmark). The A_260_*/*A_280_ ratio of ≥ 1.8 was considered as a good quality DNA. The extracted DNA was kept at − 20 °C [23].

### Molecular characterization by Polymerase Chain Reaction (PCR) and DNA sequencing

Carriage of β-lactamase genes associated with ESBL phenotype (*bla*_CTXM-15_, *bla*_CTXM-27_, *bla*_TEM*,*_ and *bla*_SHV_), and genes linked to colistin resistance (*mcr-1),* the *E. coli* O25-B2-ST131 clone (*pabB*), siderophore (*iucD*), protection proteins (*traT*), capsule (*kpsMTII*), adhesins (*afa*, *fos* and, *csgA*), and toxins (*vat*, *hlyA,* and, *cnf1*) were detected by specific primers [24–35]. The PCR product of a positive isolate containing the *pabB* was subjected to sequencing (Macrogen, Korea) to verify the authenticity of the amplicons. The result of the sequence was aligned with corresponding sequences in the GenBank database used at the national center for biotechnology information (NCBI) BLAST program (http://blast.ncbi.nlm.nih.gov/Blast.cgi? program = blastn and PAGE-TYPE = blast search and LINK-LOC = blasthome). PCR was performed according to the method provided by Moradi and Moghaddam et al. [36, 37].

### Genotyping by Repetitive Extragenic Palindromic-PCR (rep-PCR)

A total of 120 isolates were chosen for rep-PCR typing based on differences in adhesins, antibiotic resistance pattern, toxins, protection proteins, siderophore, and capsule genes using REP primer. The rep-PCR was performed according to the protocol of Arabestani el al. [38]. Phylogenetic group analysis for all isolates was accomplished based on electrophoresis results and isolates were classified into different groups.

### Statistical analysis

The REP band patterns were compared by Dice and unweight paired group (UPGMA) method and clustered using the inslico.ehu.es online databases. We determined the differences of rep-PCR profiles with the adhesins, toxins, antibiotic resistance pattern, siderophore, capsule, and protection proteins of the isolates after fingerprinting by rep-PCR. The information of each isolate was assessed in SPSS-22 software and interpretation of the results was based on the frequencies. A *p* value < 0.05 was considered statistically significant for the associations between studied variables using the Chi-square test (X^2^).

## Results

A total of 120 *E. coli* strains was isolated from urine samples of 2 to 7 years old children (102 girls and 18 boys). The isolates belonged to O25-B2-ST131 (31, 25.83%) and non-O25-B2-ST131 (89, 74.16%) and *E. coli* strains. Resistance to at least one antibiotic class was detected in 97.5% of these isolates, while three isolates were susceptible to all the antibiotics. Results of disk diffusion showed the highest resistance and the lowest resistance to ampicillin (92, 76.6%) and imipenem (5, 4.1%), respectively. Resistance to ceftazidime, cefepime, cefotaxime, cefoxitin, cefazolin, tetracycline, and trimethoprim + sulfamethoxazole was over 50% (Table 1). Statistical analysis showed that resistance to some antibiotics, such as ampicillin, cefazolin, cefoxitin, cefotaxime, ceftazidime, cefepime, ciprofloxacin, imipenem, nalidixic acid, gentamicin, aztreonam, tetracycline, amoxicillin-clavulanic acid, and trimethoprim + sulfamethoxazole were significantly higher in the strains belonged to the O25-B2-ST131 clone (*p* value < 0.05). Details of the diffusion disk results for *E. coli* isolates can be seen in Table 1.

**Table 1:** Antibiotic resistance patterns of clinical isolates of *E.coli* from UTI

Antibiotic	Total, *n* = 120		O25-B2-ST131, *n* = 31		Non- O25-B2-ST131 *n* = 89		***P*-**value
	S	R	S	R	S	R	
Azitromycin	94(78.3)	26(21.7)	28(90.3)	3(9.7)	66(74.15)	23(25.8)	0.039
Ampicillin	28(23.3)	92(76.6)	2(6.4)	29(93.5)	26(29.2)	63(70.8)	0.013
Cefazolin	42(35)	78(65)	4(12.9)	27(87.1)	38(42.7)	51(57.3)	0.008
Cefoxitin	51(42.5)	69(57.5)	9(29)	22(71)	42(47.2)	47(52.8)	0.029
Cefotaxime	44(37.7)	76(67.3)	3(9.7)	28(90.2)	41(46)	48(53.9)	0.005
Ceftazidime	44(36.7)	76(63.3)	7(22.6)	24(77.4)	37(41.6)	52(58.4)	0.004
Cefepime	49(40.8)	71(59.2)	6(19.3)	25(80.7)	43(48.3)	46(51.6)	0.008
Ciprofloxacin	63(52.5)	57(57.5)	10(32.25)	21(67.7)	53(59.5)	36(40.4)	< 0.001
Imipenem	115(95.8)	5(4.1)	30(96.7)	1(3.2)	85(95.5)	4(4.4)	0.764
Nalidixic acid	80(66.6)	40(33.3)	21(67.7)	10(32.2)	59(66.3)	30(33.7)	0.129
Gentamicin	67(55.8)	53(44.1)	12(38.7)	19(61.2)	55(61.8)	34(38.1)	0.175
Amikacin	102(85)	18(15)	28(90.3)	3(9.67)	74(83.1)	15(16.7)	0.661
Aztreonam	64(53.3)	56(46.6)	15(48.4)	16(51.5)	49(55)	40(44.9)	0.001
Tetracycline	35(29.2)	85(70.7)	8(25.8)	23(74.2)	27(30.3)	62(69.6)	< 0.001
Ampicillin sulbactam	66(55)	54(44.9)	18(58)	13(41.9)	48(53.9)	41(46)	0.415
Amoxicillin-clavulanic acid	51(42.5)	69(57.4)	10(32.2)	23(67.7)	41(46)	48(53.9)	0.038
Piperacillin tazobactam	73(60.8)	47(39.1)	22(71)	9(29)	51(57.3)	38(42.6)	0.399
Meropenem	114(95)	6(4.9)	28(90.3)	3(9.67)	86(96.6)	3(3.3)	0.679
Nitrofurantoin	90(75)	30(25)	26(83.8)	5(16.1)	64(71.9)	25(28)	0.532
Trimethoprim + Sulfamethoxazole	46(38.3)	72(61.6)	9(29)	22(70.9)	37(41.5)	52(58.4)	0.049

**Fig. 1:**
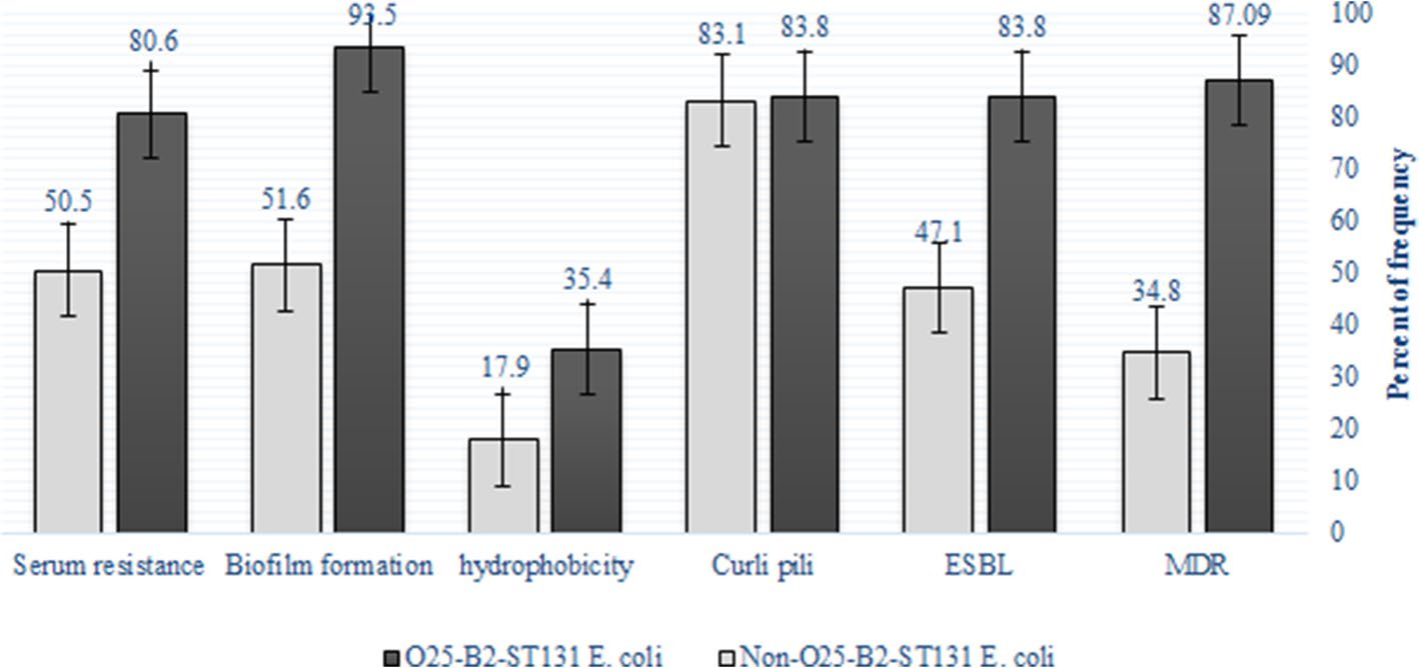
Differences in phenotypic factors involved in virulence and antibiotic resistance between O25-B2-ST131 and non-O25-B2-ST131 strains.* = Indicates the significant level of difference *P* < 0.05; ** = Indicates the significant level of difference value *P* < 0.001

Fifty eight (48.3%) isolates were considered as MDR, among which the frequency of MDR isolates was higher in O25-B2-ST131 strains. The prevalence of MDR isolates among O25-B2-ST131 and non-O25-B2-ST131 strains were 87.09% (*n* = 27/31) and 34.8% (*n* = 31/89), respectively. A significant correlation was observed between MDR isolates and O25-B2-ST131 strains as compared to the non-O25-B2-ST131 strains (*p* value < 0.05). After MIC, it was found that there were no colistin-resistant isolates. Sixty-eight (56.6%) isolates were ESBL-producing isolates. The frequency of ESBL was higher in O25-B2-ST131 strains and there was a significant association between ESBL production and O25-B2-ST131 strains (*p* value < 0.05). There was also a significant association between MDR isolates and ESBL production (*p* value < 0.05). The O25-B2-ST131 strains were considered as the group with higher rates of antibiotic resistance to several antibiotics such as betalactams which can be explained by the higher rate of ESBL-producing isolates in this group. Abundance of curli pili was observed in 100 (83.3%) isolates. Although the frequency of curli pili in O25-B2-ST131 strains (83.8%) was higher than non-O25-B2-ST131 strains (83.1%), there was no significant relationship between curli pili and O25-B2-ST131 strains (*p* value > 0.05). In addition, 27 (22.5%) isolates showed hydrophobicity, of which 11 (40.7%) O25-B2-ST131 and 16 (59.2%) non-O25-B2-ST131 strains had hydrophobicity. A total of 53 isolates were capable of hemolysis, of which 11 (20.7%) and 42 (79.2%) strains were O25-B2-ST131 and non-O25-B2-ST131, respectively. On one side, 70 isolates (58.3%) showed the ability to resist the bactericidal properties of serum and on other side, 75 (62.5%) isolates had the ability of biofilms formation (15 weak biofilm isolates, 23 intermediate biofilm isolates, and 37 strong biofilm isolates). In O25-B2-ST131 *E. coli*, 17 (54.8%), 9 (29%), and 3 (9.6%) strains were able to form strong, intermediate, and weak biofilms, respectively. In contrast, in non-O25-B2-ST131 *E. coli*, 20 (22.4%), 14 (15.7%), and 12 (13.4%) strains showed the ability to form strong, intermediate, and weak biofilms, respectively. These studied phenotypic characteristics showed that the level of serum resistance, bactericidal resistance (80.6% vs. 50.5%), and biofilm formation (93.5% vs. 51.6%) was significantly higher among O25-B2-ST131 compared with non- O25-B2-ST131 strains (*p* value ≤ 0.05) (Fig. 1). Among 58 MDR isolates, 45 (77.5%) isolates had the potential for biofilm formation and there was a significant relationship between biofilm formation and MDR (*p* value < 0.05).

**Fig. 2:**
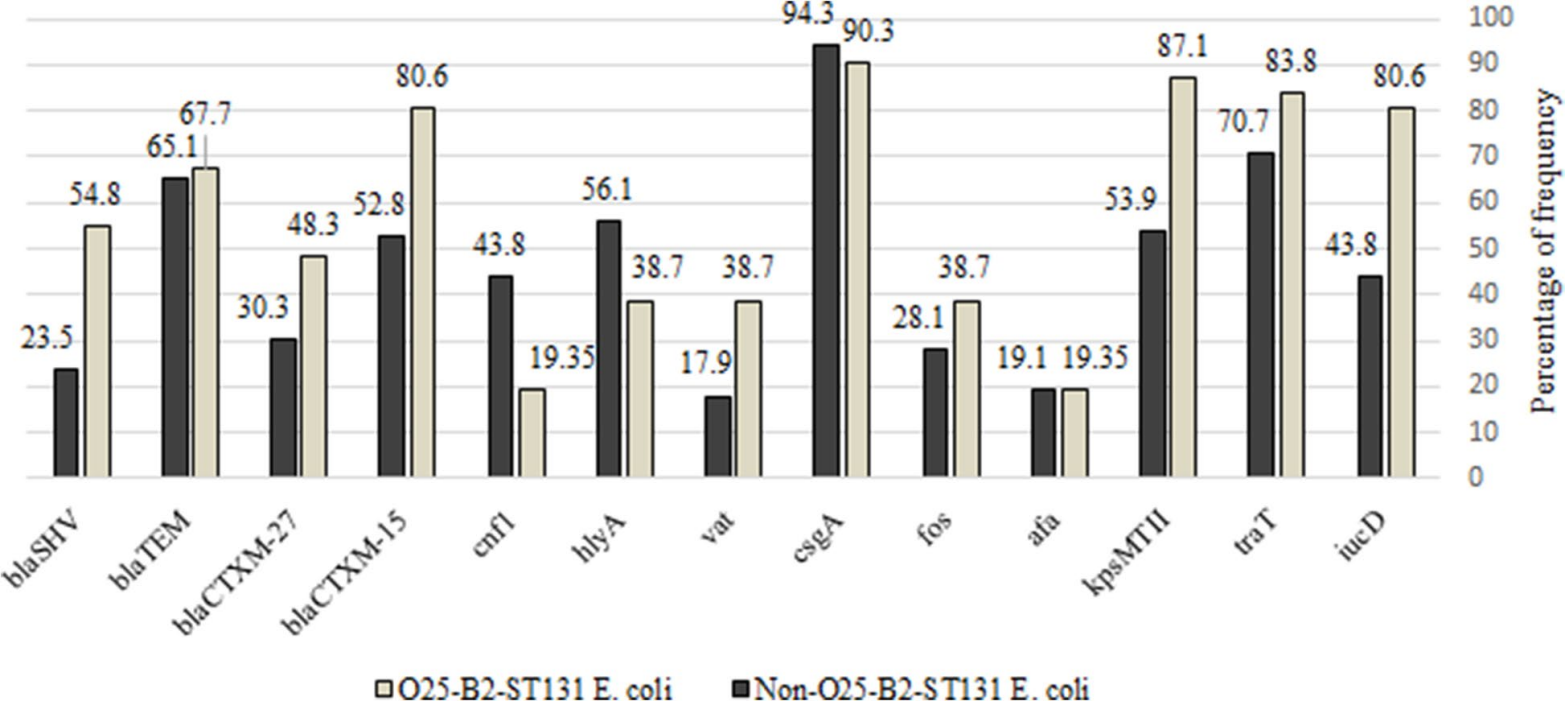
Comparison of the frequency of antibiotic resistance and virulence genes in O25-B2-ST131 and non-O25-B2-ST131 strains

**Fig. 3:**
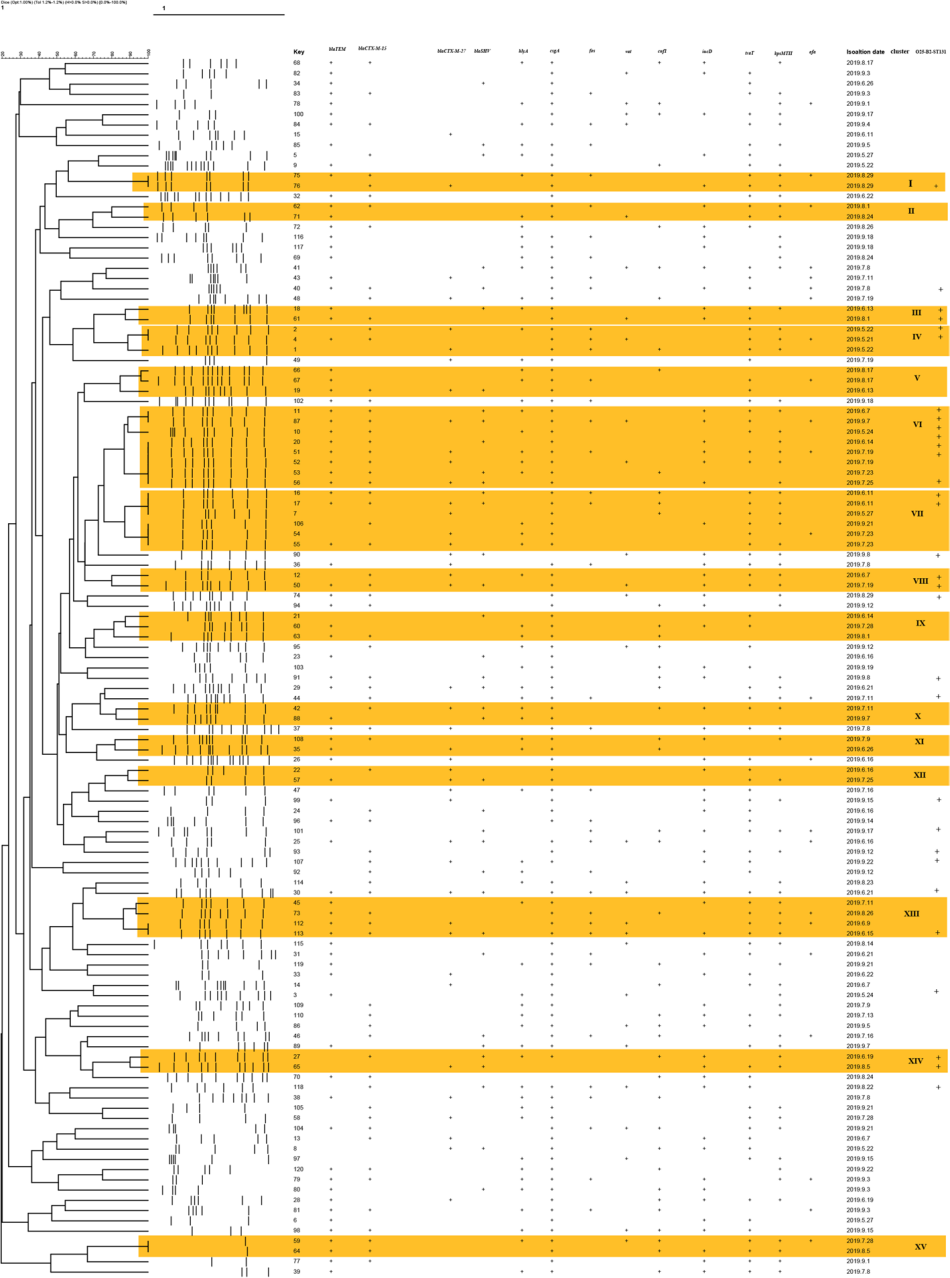
Dendrogram analysis of rep-PCR fingerprint for 120 *E. coli* isolates;** + **: Indicates the presence of antibiotic resistance or virulence genes; Clusters were highlighted in orange; Key: indicates the isolate number

Genotypic analysis of antibiotic resistance and virulence genes among 120 isolates by PCR showed that the abundance of *afa, kpsMTII, traT, iucD, Cnf1, vat, fos, csgA, hlyA, bla*_*SHV*_*, bla*_*CTXM-27*_*, bla*_*CTXM-15*_, and *bla*_*TEM*_ genes were 23 (19.2%), 75 (62.5%), 89 (74.2%), 64 (53.3%), 45 (37.5%), 28 (23.3%), 37 (30.8%), 112 (93.3%), 62 (51.6%), 38 (31.7%), 42 (35.0%), 72 (60.0%), and 79 (65.8%), respectively. The prevalence of different virulence genes in O25-B2-ST131 in comparison with non-O25-B2-ST131 *E. coli* were as follows: *iucD* (*n* = 25; 80.6% *vs n* = 39; 43.8%), *traT* (*n* = 26; 83.8% *vs n* = 63;70.7%), *kpsMTII* (*n* = 27; 87.1% *vs* *n* = 48; 53.9%), *afa* (*n* = 6; 19.35% *vs n* = 17; 19.1%), *fos* (*n* = 12; 38.7% *vs n* = 25; 28.1%), *csgA* (*n* = 28; 90.3% *vs* *n* = 84; 94.3%), *vat* (*n* = 12; 38.7% *vs n* = 16; 17.9%), *hlyA* (*n* = 12; 38.7% *vs n* = 50; 56.1%), and *cnf1* (*n* = 6; 19.35% *vs n* = 39; 43.8%). Statistical analysis showed that among the studied virulence genes, there was a significantly high number of *iucD, kpsMTII, cnf1,* and *vat* genes in the O25-B2-ST131 strains (*p* value < 0.05). In total, 72 (60.0%), 42 (35%), 79 (65.8%), and 38 (31.7%) isolates carried *bla*_CTXM-15_, *bla*_CTXM-27_*, bla*_*TEM*_, and *bla*_SHV_ genes, respectively. The prevalence of resistance genes among O25-B2-ST131 strains were as follow: *bla*_CTXM-15_ *(n* = 25; 80.6%), *bla*_CTXM-27_ (*n* = 15; 48.3%), *bla*_TEM_ (*n* = 21; 67.7%), and *bla*_SHV_ (*n* = 17; 54.8%), but in non-O25-B2-ST131 strains the prevalence was as follows: *bla*_CTXM-15_ (*n* = 47; 52.8%), *bla*_CTXM-27_ (*n* = 27; 30.3%), *bla*_TEM_ (*n* = 58; 65.1%), and *bla*_SHV_ (*n* = 21; 23.5%) (Fig. 2). Although the frequency of all four antibiotic resistance genes was higher in O25-B2-ST131 compared to the non-O25-B2-ST131 strains, frequencies of the *bla*_CTXM-15_ *and bla*_SHV_ genes in O25-B2-ST131 isolates were significantly higher in non-O25-B2-ST131 strains (*p* value ≤ 0.05). Examination of plasmid-borne colistin resistance genes showed that none of the isolates carried the *mcr*-1 gene. This research revealed that O25-B2-ST131 and non-O25-B2-ST131 strains are located in fifteen genetic clusters with 80% homology (Fig. 3). The largest cluster consisted of eight strains, seven of which belonged to the O25-B2-ST131 isolate. Among 31 O25-B2-ST131 strains, 19 isolates were classified as various clusters. Comparison of strains in the same cluster showed that some virulence and resistance genes are present in homologous variants with rep-PCR patterns. Sixteen and twelve patterns of resistance genes were observed in non-O25-B2-ST131 and O25-B2-ST131 strains, respectively (Table 2). The most predominant pattern was found in 26 isolates with the pattern of UP7, characterized by the *bla*_CTXM-15_ and *bla*_TEM_ genes, whereas two isolates (non-O25-B2-ST131) had no resistance genes (UP16 pattern). The phylogenetic analysis showed that the most common strains responsible for ESBL-producing *E. coli* isolates belonged to the phylogenetic groups B2. Also, 100% (*n* = 31/31) isolates of *E. coli* O25-B2-ST131 and 78.65% (*n* = 70/89) of non-O25-B2-ST131 belonged to group B2. Although there was not phylogenetic groups such as A, B1, and D in the O25-B2-ST131 isolates, these serotypes were found in 5.6% (*n* = 5/89), 2.24% (*n* = 2/89), and 13.48% (*n* = 12/89) of non- O25-B2-ST131 isolates, respectively.

**Table 2:** Diversity of ESBL encoding *E. coli* isolates in urine samples of children with community-acquired urinary tract infection in Tehran, Iran

Pattern (UP)	Number of isolates			Resistance genes			
	**Total** **(*N* = 120)**	**Non- O25-B2-ST131** **(*N* = 89)**	**O25-B2-ST131** **(*N* = 31)**				
				***bla***_**TEM**_	***bla***_**CTX-M-15**_	***bla***_**CTX-M-27**_	***bla***_**SHV**_
1	8 (6.6%)	4 (4.4%)	4 (12.9%)	-	+	+	-
2	7 (5.8%)	7 (7.8%)	0	-	-	+	-
3	17 (14.1%)	16 (17.9%)	1 (3.2%)	+	-	-	-
4	9 (7.5%)	8 (8.9%)	1 (3.2%)	+	-	+	-
5	3 (2.5%)	1 (1.1%)	2 (6.4%)	-	-	+	+
6	9 (7.5%)	7 (7.8%)	2 (6.4%)	-	+	-	-
7	26 (21.6%)	22 (24.7%)	4 (12.9%)	+	+	-	-
8	8 (6.6%)	4 (4.4%)	4 (12.9%)	+	+	-	+
9	7 (5.8%)	2 (2.2%)	5 (16.1%)	+	+	+	+
10	7 (5.8%)	6 (6.7%)	1 (3.2%)	+	-	-	+
11	3 (2.5%)	1 (1.1%)	2 (6.4%)	-	-	-	+
12	7 (5.8%)	5 (5.6%)	2 (6.4%)	-	+	-	+
13	5 (4.1%)	2 (2.2%)	3 (9.6%)	+	+	+	-
14	1 (0.8%)	1 (1.1%)	0	-	+	+	+
15	1 (0.8%)	1 (1.1%)	0	+	-	+	+
16	2 (1.6%)	2 (2.2%)	0	-	-	-	-

+  Indicates the presence of antibiotic resistance gene in the strain, while - indicates the absence of antibiotic resistance gene in the strain

## Discussion

*E. coli* ST131 is a universal clone of antimicrobial-resistant *E. coli* isolated in most clinical samples [39]. In the current study, based on the presence of *pabB* gene using allele-specific PCR, 31 (25.83%) strains were subsequently assigned as O25-B2-ST131 *E. coli*. However, in line with the results of our study, Rasoulinasab et al. reported a prevalence of 26.9% of *E. coli* O25b/ST131 in patients with urinary tract infection in Iran [40]. However, the ST131 outbreak has occasionally been reported around the world, including Japan (10%), Denmark (38%) [41], and Australia (51%) [42]. Various research reports have inconsistencies that can be related to differences in the study population, sample size, age groups (children versus the elderly), type of samples (urine versus a diverse range of clinical specimens), and detected O-serogroups [43]. Our result showed that in antibiogram test the highest resistance was against ampicillin and the lowest resistance was against imipenem.‌ Similar to the results of our study, studies have shown that resistance to ampicillin is the most common among antibiotics, but resistance to imipenem is very low [39, 44]. As a result, to prevent the incidence of hospital and community infection outbreaks, investigating the international distribution of *E. coli* ST131 could be a helpful strategy. In the present research, the prevalence rate of ampicillin resistance in *E. coli* was the highest. The observed resistance pattern to ampicillin in *E. coli* ST131 clones in the current research was consistent with former studies in different provinces of Iran [45, 46]. The highest prevalence of ampicillin resistance was found in isolates of patients ≤ 12 years old, and resistance rate to this antibiotic was low in the current study [47]. Another study in the USA indicated that *E. coli* ST131 clones had a resistance rate of 97.8% to ampicillin [48]. In the United Kingdom, high levels of ampicillin resistance (55%) were found in isolates of *E. coli* ST131 clones and are consistent with the findings of our study [49]. Interestingly, the antibiotic resistance rate was higher in the O25-B2-ST131 strains in comparison with non-O25-B2-ST131 strains. Consistent with our results, previous studies reported a high frequency of antibiotic resistance in O25-B2-ST131 strains which turned them into a clinical challenge [40, 44, 50]. The MIC results showed that none of the isolates, even *E. coli* O25-B2-ST131 strains, were resistant to colistin, and colistin was the most effective antibiotic against *E. coli* isolates from UTI. Although a small number of colistin-resistant *E. coli* ST131 strains have been isolated from different samples in studies, colistin has still been identified as the most effective antibiotic in previous studies [2, 51]. Altogether, colistin is probably more effective than other antibiotics in treating UTIs. However, the nephrotoxic properties of this antibiotic limit its usage as the drug of choice for the treatment of urinary tract infections [52]. In our study, not only the frequency of MDR was high among 120 isolates (48.3%) but also the prevalence of MDR among O25-B2-ST131 strains (87.09%) was higher than non-O25-B2-ST131. *E. coli* ST131 clone as an MDR pathogen that has recently been considered a huge public health issue. In line with our study, it was found that the rate of MDR in *E. coli* ST131 is high as one of the high risk clones and is defined as one of the clones with a global distribution which has a high ability to survive, clone and spread in different types of niches. On the other hand, *E. coli* ST131 has been identified as an O25b: H4 serotype and the highly dangerous phylogenetic group B2, which carries high amounts of MDR IncFII plasmids containing *bla*_*CTXM-15*_ [53, 54]. In total, 56.6% of isolates were ESBL producers and ESBL production was more common in O25-B2-ST131 isolates compared to the non-O25-B2-ST131 isolates. In a study, 31–36% of *E. coli* strains produced ESBL in Korea and in consistent with our study, ST131 isolates were significantly associated with ESBL, specifically CTX-M-15, and were mostly MDR [55]. This suggests that the frequency of ESBL can vary in different geographical regions in O25-B2-ST131 strains. Phenotypic characteristics play an essential role in the pathogenicity of UTIs caused by *E. coli*. Also, the determination and analysis of these characteristics seem to be necessary for epidemiological studies. The O25-B2-ST131 isolates formed a more robust biofilm compared to the non-O25-B2-ST131 isolates. This result is in accordance with the study by Mostafavi et al. [50]. The potency of *E. coli* to evade the bactericidal effect of serum compounds, such as complement and antimicrobial peptides, makes it an advantage for extraintestinal *E. coli* that enters the bloodstream. Serum bactericidal resistance was mostly observed in O25-B2-ST131 isolates compared with the non-O25-B2-ST131 isolates. Previous results reported high levels of serum bactericidal resistance among O25-B2-ST131 strains [50, 56]. Consistent with our study, Duprllot et al. reported high rates of curli production in O25-B2-ST131 isolates [57]. Olsen et al. showed that curli is not produced by the most pathogenic *E. coli* strains when grown at 37 °C [58]. One explanation for this contradiction in several studies may be the genetic differences among strains and growth conditions (media and temperature) [59]. As a high-risk pandemic strain, *E. coli* sequence type (ST) 131 has been identified in human, food, environmental, and animal samples. *E. coli* ST131 has been repeatedly reported to carry clinically important antimicrobial resistance genes and is associated with extraintestinal diseases, mainly UTI. In this study, except for *mcr-1* gene, other virulence genes including *iucD*, *traT*, *kpsMT11*, *afa*, *fos*, *csgA*, *vat*, *hlyA*, and *cnf1* were detected in O25-B2-ST131 and non- O25-B2-ST131 isolates. It was also found that the frequency of the majority of virulence factors in O25-B2-ST131 was higher compared to non-O25-B2-ST131 strains, which indicates the high pathogenicity of O25-B2-ST131. As with our results, previous studies have agreed that the virulence factors of O25-B2-ST131 strains are significant as a dangerous clone type and that these strains are considered as a therapeutic challenge [40, 50]. Although the frequency of *mcr* genes in *E. coli* ST131 strains is very low, studies have reported the prevalence of this gene in *E. coli* ST131 strains isolated from animal and environmental samples [60, 61]. In contrast to our study, some studies have confirmed the presence of the *mcr* gene in *E. coli* strains isolated from human samples in America, Japan and Korea which indicates that the frequency of these plasmid genes can be different based on the isolation source and geographical environment [62–64]. On the other hand, *E. coli* isolates were found to carry antibiotic resistance genes such as *bla*_*CTXM-15*_, *bla*_*CTXM-27*_*, bla*_*TEM*_, and *bla*_*SHV*_ genes. The abundance of these important genes in antibiotic resistance was higher in O25-B2-ST131 strains compared to non-O25-B2-ST131 strains. Antibiotic resistance studies have been performed in O25-B2-ST131 strains which confirm our results. These studies have shown that resistance and the presence of genes involved in resistance are higher in O25-B2-ST131 strains [40, 50, 62]. It is also shown that *E. coli* ST131 has a wide range of virulence and resistance genes located on plasmids with high transmissibility that has a global spread [39]. In preceding studies, the O25-B2-ST131 *E. coli* with resistance genes and high virulence potential has been described worldwide. A worrying elevation in the isolation of *E. coli* isolates with the ability to produce CTX-M-15 from different countries has been described, and this phenomenon is associated with the development of the clonal ST131 [65, 66]. Resistance in *E. coli* ST131 has been commonly reported worldwide and associated with other resistance genes [45]. In previous studies, the percentage of virulence factors among ST131 strains has been reported with occasional variations [65, 67]. Although in the above studies different virulence genes were usually investigated in O25-B2-ST131 *E. coli* strains, the important point is that the amount of antibiotic resistance and virulence genes in these strains is high. Examination of all characteristics of *E. coli* isolates from children with UTI revealed that phylogenetic group B2 was the most common in all isolates (*n* = 101; 84.16%) and O25-B2-ST131 strains. Consistent with our study, Hojabri et al. showed that the existent ST131 strains were considerably more similar to the B2 group than the *E. coli* and non-ST131 isolates [68]. In other studies, the high prevalence of B2 group was reported among UPEC isolates that are well known worldwide [69, 70]. The higher prevalence of group B2 among UPEC isolates is due to the antibiotic resistance genes and virulence factors existing within this group which can cause an increased survival fitness in the urinary tract [70]. Another part of our results showed that O25-B2-ST131 and non-O25-B2-ST131 strains are located in fifteen different genetic clusters with 80% homology. The largest cluster consisted of eight strains, seven of which belong to the O25-B2-ST131 isolate. Of 31 O25-B2-ST131 isolates, 19 isolates were classified as various clusters. According to our study, several specific host sub-clusters were found in the McLellan study. Approximately 33% of the strains showed less than 65% similarity [71].

The present study encountered several limitations. The study was done in one hospital, and due to budget limitation doing a multicenter study was not possible. Access to medical data of the children, like underlying diseases and recent medications, to correlate their links with colonization of *E. coli* O25-B2-ST131 strains and their antimicrobial resistance phenotypes was not possible, because of the lack of a registry system for outpatients. Moreover, no follow-up program at the time of study was considered to understand differences in the success or complications of UTI by *E. coli* O25-B2-ST131 strains in comparison to non-O25-B2-ST131 strains. Hence, many mechanisms and resistance genes that can prove higher resistance in *E. coli* O25-B2-ST131 strains were not investigated such as carbapenems, aminoglycosides, and fluoroquinolones due to the costs getting higher in this study.

In conclusion, obtained results showed a higher frequency of antibiotics resistance and virulence factors in O25-B2-ST131 strains compared with other *E. coli* isolates in children with CA-UTI. The high frequency of antibiotic resistance and virulence genes in O25-B2-ST131 strains, which can be the cause of increased pathogenicity and treatment failure, showed the importance of these strains in the children's infections. In this study, *E. coli* isolates with common rep-types presented a diversity in their clone types, virulence capacity and antibiotic-resistance patterns. Constant monitoring, due to the high prevalence of these strains and their involvement in UTI, should be done to control their spread in the community.

## Data Availability

All data supporting the conclusions of this article are included within the article. The *pab*B gene sequence of the strains was deposited in the GenBank under accession number OK356605.1. (https://www.ncbi.nlm.nih.gov/nuccore/OK356605.1)

## Abbreviations

UPEC: Uropathogenic *Escherichia coli*
UTI: Urinary tract infection
MIC: Minimum growth inhibitory concentration
